# Experimental duplication of bilaterian body axes in spider embryos: Holm’s organizer and self-regulation of embryonic fields

**DOI:** 10.1007/s00427-019-00631-x

**Published:** 2019-04-10

**Authors:** Hiroki Oda, Sawa Iwasaki-Yokozawa, Toshiya Usui, Yasuko Akiyama-Oda

**Affiliations:** 1 Laboratory of Evolutionary Cell and Developmental Biology, JT Biohistory Research Hall, 1-1 Murasaki-cho, Takatsuki, Osaka 569-1125 Japan; 2 Department of Biological Sciences, Graduate School of Science, Osaka University, Toyonaka, Osaka Japan; 3 Nagoya Minami High School, Nagoya, Aichi Japan; 4 Microbiology and Infection Control, Osaka Medical College, Takatsuki, Osaka Japan

**Keywords:** Axis specification, Spemann organizer, Chordin, Arthropod evolution, Model organism, *Parasteatoda tepidariorum*

## Abstract

Bilaterally symmetric body plans of vertebrates and arthropods are defined by a single set of two orthogonal axes, the anterior-posterior (or head-tail) and dorsal-ventral axes. In vertebrates, and especially amphibians, complete or partial doubling of the bilaterian body axes can be induced by two different types of embryological manipulations: transplantation of an organizer region or bi-sectioning of an embryo. Such axis doubling relies on the ability of embryonic fields to flexibly respond to the situation and self-regulate toward forming a whole body. This phenomenon has facilitated experimental efforts to investigate the mechanisms of vertebrate body axes formation. However, few studies have addressed the self-regulatory capabilities of embryonic fields associated with body axes formation in non-vertebrate bilaterians. The pioneer spider embryologist Åke Holm reported twinning of spider embryos induced by both types of embryological manipulations in 1952; yet, his experiments have not been replicated by other investigators, and access to spider or non-vertebrate twins has been limited. In this review, we provide a historical background on twinning experiments in spiders, and an overview of current twinning approaches in familiar spider species and related molecular studies. Moreover, we discuss the benefits of the spider model system for a deeper understanding of the ancestral mechanisms of body axes formation in arthropods, as well as in bilaterians.

**Electronic supplementary material:**

The online version of this article (10.1007/s00427-019-00631-x) contains supplementary material, which is available to authorized users.

## Introduction

The bilaterally symmetric body pattern of “bilaterian” animals is typically defined by a single set of two orthogonal body axes: the anterior-posterior (AP) or head-tail axis and the dorsal-ventral (DV) axis. Vertebrates and arthropods are two representative monophyletic groups of bilaterian animals that are phylogenetically distant from each other. Some signs of differences between bilaterian and non-bilaterian metazoans are detectable in their genomes (Putnam et al. 2007; Matus et al. 2008; Nichols et al. 2012; Hulpiau and Van Roy 2010; Fahey and Degnan 2010; Ryan et al. 2013; Simakov et al. 2013; Moroz et al. 2014; Sasaki et al. 2017; Paps and Holland 2018) and denote an evolutionary shift from non-bilaterians to bilaterians. Reconstructing the putative genome, morphology, and patterning system present in the last common ancestor of vertebrates and arthropods, or of all bilaterians, represents an ongoing effort in evolutionary biology.

Bilaterian body axes in vertebrate embryos can be totally or partially duplicated following certain embryological manipulations. Historically, Hans Spemann induced formation of twins by ligating newt eggs (Spemann 1901; Sander and Faessler 2001) and then, with Hilde Mangold, by transplanting the dorsal lip of an early newt gastrula embryo to the ventral side of another embryo (Spemann and Mangold 1924). These two different types of classical embryological axes-doubling experiments have exposed two key concepts in developmental biology: self-regulation and organizer (Spemann, 1938; Sander and Faessler 2001; De Robertis 2009). Since the advent of modern molecular genetics, investigators’ efforts using several vertebrate models, especially the amphibian *Xenopus laevis*, have successfully incorporated classical embryological techniques and findings to identify genes, molecules, their interactions, and mechanisms of their actions that substantiate the organizer and self-regulation concepts for vertebrate body axes formation (De Robertis 2009).

Nevertheless, it has remained unclear whether these concepts could be applied to body axes formation in other bilaterians, such as arthropods. This lack of knowledge has limited our understanding of the earliest mechanism that allowed animals to diversify their strategies to form bilaterally symmetric body plans. The main aim of this review is to promote investigation into self-regulation mechanisms associated with body axes formation in arthropod cell-based embryonic fields by introducing the spider model system. Spiders are phylogenetically distant from the popular model insects within Arthropoda (Rota-Stabelli et al. 2013). A secondary aim is to discuss the practical benefits of spider model systems for analyzing embryological mechanisms at cellular resolution. Although molecular details about body axes formation are available from a broad phyletic range of metazoan animals, they are beyond the scope of this article (see Lynch and Roth 2011; Bier and De Robertis 2015; Genikhovich and Technau 2017). Here, we focus on experimentally induced embryological phenomena that are similar or analogous between vertebrates and spiders and which may rely on both local and long-distance cell-cell interactions.

## Holm’s organizer in spider embryos

In 1952, Åke Holm (Kronestedt 1989) reported a comprehensive set of embryological experiments using the funnel-web spider species *Agelena labyrinthica* (Holm 1952). The experiments included fate mapping, extirpation, and transplantation. A typical obstacle in manipulating arthropod embryos is the vitelline membrane, which usually cannot be removed without impairing embryo development. To overcome this obstacle, he designed and constructed an original micropipette, called piston micropipette, which allowed him to manipulate embryos through a small break introduced in the vitelline membrane.

**Fig. 1:**
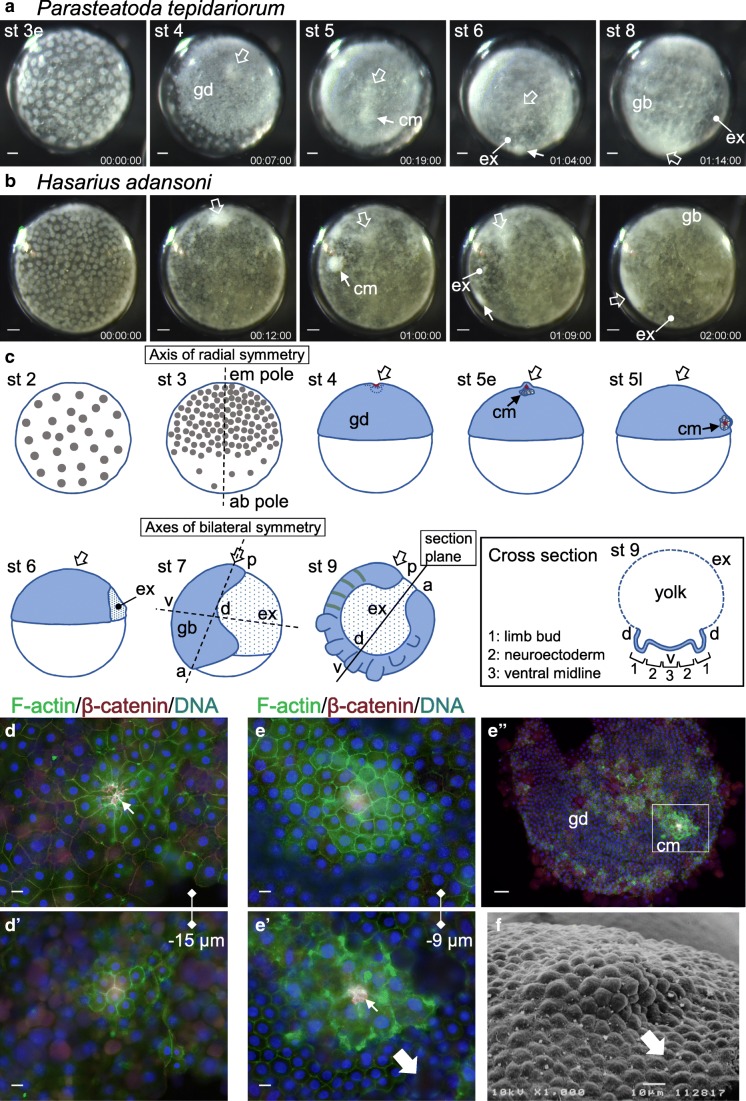
Development and cellular characterization of the cumulus in spider embryos. (A, B) Time-lapse observation of wild-type *Parasteatoda tepidariorum* (A) and *Hasarius adansoni* (B) embryos. The relative time (days: hours: minutes) and stages (st), including early (e) and late (l) stages, are indicated. Wide open arrows indicate the embryonic pole or subsequent posterior terminal region of the embryo. Solid arrows denote the cumulus (cm). The cumulus forms at the embryonic pole of the early embryo, after which it shifts to an equatorial area, where the extraembryonic cell fate (ex) is specified. The cumulus in *H. adansoni* shows a denser cell mass than that in *P. tepidariorum*. Images shown in (A) are adapted from (Akiyama-Oda and Oda 2010). The positions of the germ disc (gd) and germ band (gb) are indicated. The embryo shown in (B) is the same as the left embryo in Movie S2. (C) Schematic representation of *P. tepidariorum* embryos at various stages, highlighting the emergence of body axes. Embryos are viewed from the lateral side except in the inset, where a stage 9 embryo is cross-sectioned as indicated. Broken black lines indicate the axis of radial symmetry, which runs through the embryonic (em) and ab-embryonic (ab) poles, as well as the axes of bilateral symmetry: the anterior (a)-posterior (p) and dorsal (d)-ventral (v) axes. β-Catenin at the forming and formed cumulus is indicated in red, with the inner materials of the cumulus depicted. (D, E) Selected slices of three-dimensional image stacks showing the cellular organization of the embryonic polar region in the germ disc-forming stage embryo (D, D’) and that of the cumulus in the cumulus-shifting stage embryo (E, E’) in *P. tepidariorum*. Embryos were triple-stained for F-actin (green), β-catenin (red), and DNA (blue) and flat-mounted (Supplementary text). The slices shown capture the apical portion of the surface epithelium in (D) and (E) and are moved toward the inside of the embryo by 15 and 9 μm in (D’) and (E’), respectively. (E”) is a low-magnification image of the embryo, in which the boxed region corresponds to (E) and (E’). Thin arrows in (E’) indicate strong concentrations of β-catenin at adherens junctions, which appear to physically connect the cells. (F) Scanning electron microscopy image showing the cumulus, adapted from (Akiyama-Oda and Oda 2003). Wide solid arrows in (E’) and (F) indicate the direction of cumulus shifting. Bars, 50 μm in (A, B, E”); 10 μm in (D, D’, E, E’, F)

Holm reached one simple conclusion and stated “The primitive cumulus is an organizing center, which determines the dorsal axial system in a similar way to the marginal zone in the amphibian embryo” (Holm 1952). The term “primitive cumulus” indicates a small region characteristic of an early spider embryo, which was initially described in the long-legged spider *Pholcus opilionoides* (Claparede 1862) and later in other spider species using various terms such as “cumulus,” “secondary thickening,” and “posterior cumulus.” Hereafter, we will call it simply cumulus. The cumulus morphology varies among spider species (Fig. 1A, B; Wolff and Hilbrant 2011; Turetzek and Prpic 2016). In the common house spider *Parasteatoda tepidariorum*, the cumulus is observed as a slightly protruding white material, which consists of a cluster of migratory inner cells and the static surface cell layer (Fig. 1A, C–F; Akiyama-Oda and Oda 2003). The inner cells originate at the embryonic pole of a radially symmetric embryo by cell internalization through a transient blastopore (Fig. 1A, C, D) and, during migration, appear to retain dense adherens junctions formed in the preceding epithelial state (Fig. 1C–E; Oda et al. 2007). It is possible that adherens junctions prevent the migratory cells from being scattered.

The cumulus breaks the radial symmetry following the onset of movement (Akiyama-Oda and Oda 2003; Akiyama-Oda and Oda 2010; Pechmann et al. 2017). The direction of cumulus movement allows one to predict the orientation of the future AP and DV axes (Fig. 1C). The cumulus cells travel to an equatorial area of the embryo (corresponding to the rim of the germ disc in *Parasteatoda*), where extraembryonic cell fate is induced, and then the region of extraembryonic cells expands to mark the future dorsal side of the embryo, where closure will occur (Hemmi et al. 2018). In concert with this dorsal extraembryonic expansion, the embryonic cells form a germ band accompanied by convergent extension movement and cell division (Kanayama et al. 2011; Hemmi et al. 2018), with the AP and DV axes becoming morphologically evident. The germ band elongates along the AP axis, which displays morphological and molecular traits highly conserved among arthropods (Peel et al. 2005). These traits include an increasing number of segments and a bilaterally symmetric spectrum of DV pattern elements; limb buds form at the dorsal-most regions of the germ band, the ventral midline at the ventral-most (medial) region, and the neuroectoderm at the intermediate regions (Fig. 1C; Stollewerk 2002; Akiyama-Oda and Oda 2006; Linne and Stollewerk 2011).

**Fig. 2:**
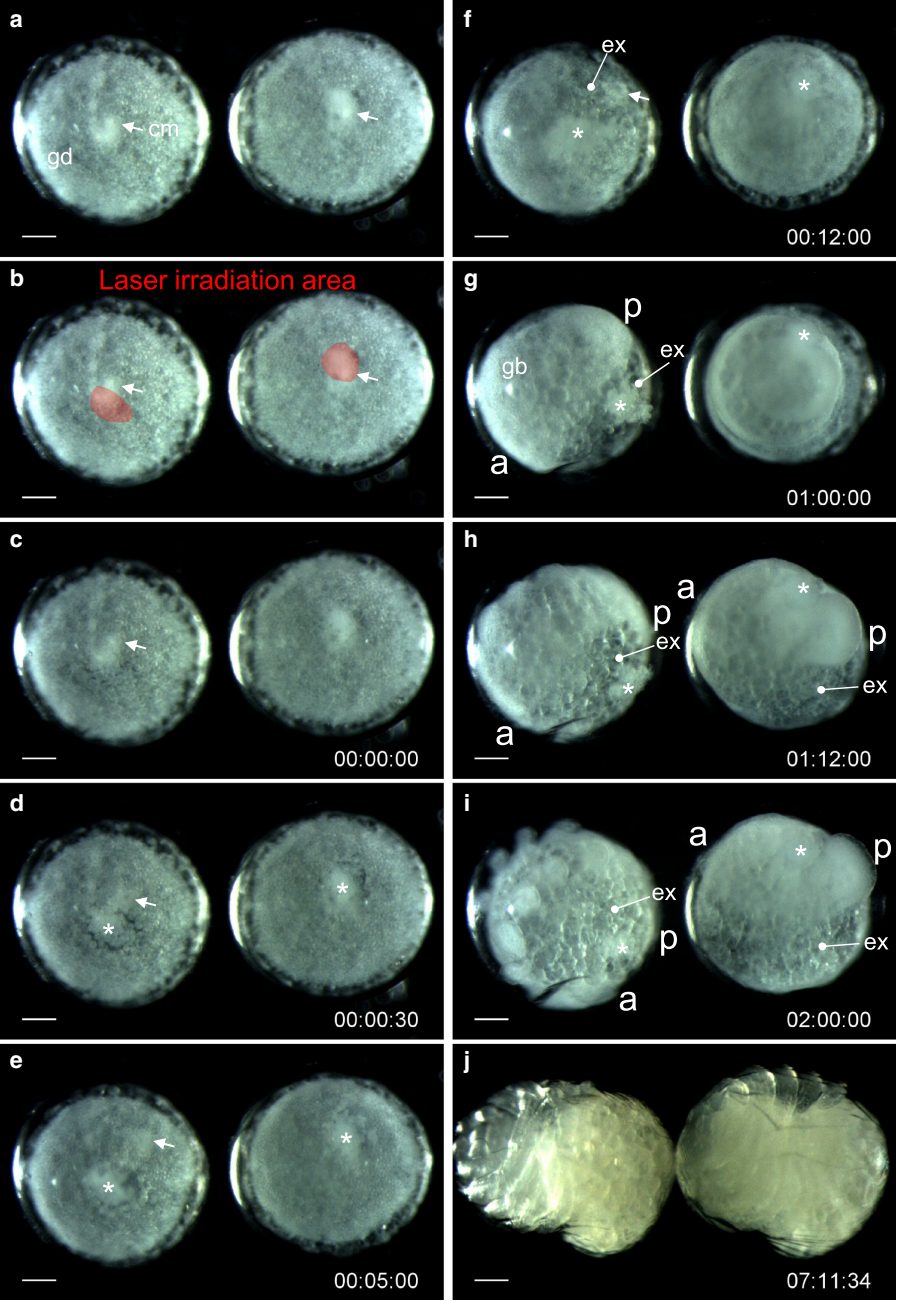
Laser-based ablation of the cumulus in *P. tepidariorum* embryos. Two embryos at the early cumulus-shifting stage are irradiated with a laser. (A, B) Embryos several minutes before laser irradiation; the corresponding irradiated area is marked in red in (B). (C–J) Time-lapse recording (days: hours: minutes) after laser irradiation (C, time 0). Arrows indicate the cumulus. The left embryo shows a visible cumulus shift to the rim of the germ disc and is seen to develop quite normally, whereas the right one shows neither a visible cumulus shift nor timely extraembryonic induction, but persistent radial symmetry. Albeit with a delay, the right embryo is later seen to develop DV asymmetry (H, I) and form a rather normal body (J). Asterisks indicate cell debris that resulted from the laser irradiation. cm, cumulus; gd, germ disc; ex, extraembryonic region; gb, germ band; a, anterior; p, posterior. Bars, 100 μm. See also Movie S1


Holm extirpated the cumulus during migration in *Agelena* embryos (Holm 1952). This manipulation resulted in embryos showing persistent radial symmetry with no typical germ band formed. Extirpation is relatively easily performed using a glass capillary, but we found that it could be replicated more effectively using a laser ablation system (Supplementary text). When the cumulus and some surrounding areas in *Parasteatoda* embryos were extirpated shortly after the start of cumulus shifting, the embryos failed to develop extraembryonic tissue at the normal timing and instead exhibited persistent radial symmetry (Fig. 2; Movie S1). Although the embryos mostly achieved a rather normal body form, the process took much longer (right embryo in Fig. 2). In control, when a similar region of similar size was extirpated but a part of the cumulus was allowed to survive, embryonic development was quite normal (left embryo in Fig. 2). These results indicate that Holm’s extirpation experiments can be partially reproduced using a different spider species.

Movie S1Laser-based ablation of the cumulus in *P. tepidariorum* embryos. Two embryos at the early cumulus-shifting stage are irradiated with a laser. The irradiated area is marked in red. Several minutes after laser irradiation, time-lapse recording starts (days: hours: minutes). The left embryo shows a visible cumulus shift to the rim of the germ disc and is seen to develop quite normally; whereas the right one shows neither a visible cumulus shift nor timely extraembryonic induction, but persistent radial symmetry. Albeit with a delay, the right embryo is later seen to develop DV asymmetry and form a rather normal body. cm, cumulus; ex, extraembryonic region; L1–L4, first to fourth walking legs; Pp, pedipalp. Bars, 100 μm. This movie is related to Fig. 2. (MP4 21477 kb)


**Fig. 3:**
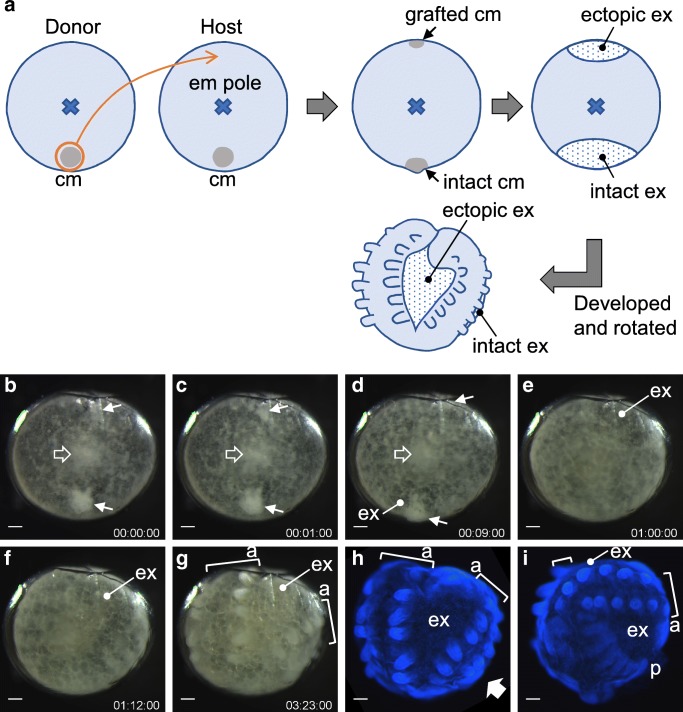
Transplantation of the cumulus between sibling embryos in *H. adansoni*. (A) Schematic outline of the experiment. A cumulus (cm) from a donor embryo (upper solid arrows in B–D) is transplanted at a site opposite the intact cumulus (lower solid arrows in B–D) with respect to the embryonic (em) pole (wide open arrows in B–D) in a host embryo, as highlighted in orange. The grafted cumulus appears to be associated with ectopic induction of extraembryonic tissue. Two sets of bilaterally symmetric body elements are formed in embryonic regions intervening between the intact and ectopic extraembryonic tissues. (B–G) Time-lapse recording (days: hours: minutes) after transplantation (B, time 0). (H, I) The recorded embryo was fixed within 5 min after image (G) was taken, and was stained for DNA. In (H), the stained embryo is viewed from an angle similar to that in (G). In (I), the same stained embryo is viewed from an angle indicated by the wide solid arrow in (H). Square brackets indicate doubled germ bands, each having bilateral pairs of the serial limb buds. ex, extraembryonic region; a, anterior; p, posterior. See also Movie S2. Bars, 50 μm


Holm transplanted a part of the cumulus to the opposite side of the same embryo. This manipulation resulted in doubling of bilaterally symmetric body patterns in a single egg, producing two separate sets of AP and DV axes. He marked grafts with a vital dye to show induction of axes doubling and the association of marked grafts with extraembryonic tissue. This experiment provided strong evidence of the cumulus acting as an organizer capable of inducing an additional set of axes defining bilateral body symmetry. The scientific value of Holm’s organizer experiment is remarkable, but the same or similar experiments have not been conducted in any later work. Therefore, we recently made efforts to obtain twins by transplanting cumuli between sibling embryos in several spider species. Our attempts were successful with a jumping spider, *Hasarius adansoni*. Siamese twins, similar to those produced by Holm, were induced by cumulus transplantation between sibling embryos (Fig. 3; Movie S2; Supplementary text). Time-lapse recording showed that a grafted cumulus appeared to induce ectopic extraembryonic tissue, and two sets of body axes were formed between the intact and ectopic extraembryonic regions. Thus, the cumulus seems to function as an organizer of bilateral symmetry defined by two body axes in several species and may be a common feature of the spiders. Holm’s organizer, however, is critically different from Spemann’s organizer in that the center of its activity associates with extraembryonic tissue, not the middle part of the bilaterally symmetric body pattern.

Movie S2Transplantation of the cumulus between sibling embryos in *H. adansoni*. (Left) Normal embryo, corresponding to the one shown in Fig. 1B. (Right) Cumulus-grafted embryo, corresponding to the one shown in Fig. 3. A cumulus from a donor embryo (graft) is transplanted at a site opposite the intact cumulus (intact) with respect to the embryonic polar region in a host embryo. After transplantation, time-lapse recording (days: hours: minutes) starts. The recorded embryo was fixed within 5 min after the last image was taken, and was stained for DNA. The stained embryo is viewed from multiple different angles, showing doubled germ bands, each having bilateral pairs of the serial limb buds developed. cm, cumulus; ex, extraembryonic region; L1-L4, first to fourth walking legs; Pp, pedipalp; Ch, chelicera. Bars, 50 μm. (MP4 14794 kb)


Horseshoe crabs, chelicerate arthropods similar to spiders, have also been used to conduct embryological experiments since Hidemiti Oka’s pioneering work in the 1930s (Oka 1937; Oka 1943). Koichi Sekiguchi, a colleague of Oka who worked both on spider and horseshoe crab embryos (Makioka 2012), suggested the presence of an early embryonic region in horseshoe crabs that corresponded to the spider cumulus (Sekiguchi 1960). He inactivated the cumulus in horseshoe crab embryos by means of electrocauterization but failed to obtain positive evidence for its role in development. Nevertheless, his experiments suggest the possibility that a polar region of early embryos, which contributes to cumulus formation, is critical for posterior development (Sekiguchi et al. 1999). Later, Tomio Itow and colleagues succeeded in inducing twinning of horseshoe crab embryos by transplanting cells at the polar region of the embryo, called “center” cells (Itow et al. 1991). However, they were unable to produce twins when using cumuli as grafts. Accordingly, center cells, not the cumulus, are believed to represent the organizing center in horseshoe crab embryos. Considering that the spider cumulus originates at the posterior pole of the early embryo, both the spider and horseshoe crab embryos might employ a similar system for organizing bilaterian body axes. It is intriguing that, as shown in the horseshoe crab study, twinning could be induced even by injection of homogenates of center cells (Itow et al. 1991).

## Induction of twinning by separation of embryonic fields in spider embryos

Since Hans Driesch’s work on sea urchin embryos in 1891 (Driesch 1891), there have been many reports from various animal species that bisection, constriction, fragmentation, or similar manipulations of embryos lead to formation of complete or partial twins, or embryos with multiple axial structures. In contrast to sea urchin blastomere separation experiments (Driesch 1891; Hörstadius and Wolsky 1936), some of the experiments with vertebrate and arthropod embryos had fields of hundreds or thousands of cells separated. A recent study using *X. laevis* clearly showed that sagittal bisection of frog embryos into left and right halves at the 4000-cell blastula stage could result in complete twins, each with bilateral symmetry (De Robertis 2006; Moriyama and De Robertis 2018). Similarly, the fragmentation of chick embryos up to the primitive streak-forming stage is followed by development of a bilaterally symmetric axial structure within each fragment (Spratt and Haas 1960). These vertebrate examples suggest that cell populations of each of the separated fragments from an embryo are able to self-regulate to form a bilaterally symmetric, whole-body pattern. This self-regulatory capability of embryonic fields associated with body axes formation appears common among vertebrates and is likely to rely on long-distance cell-cell communication.

There have been similar examples from arthropods. Classical studies reported various examples of experimentally induced twinned embryos in insects, such as dragonflies, crickets, and leaf hoppers (Seidel 1929; Krause 1934; Sander 1971; for review see Sander 1976). However, early insect embryos are constituted of syncytia until the nuclei gain genetic autonomy through cellularization following many rounds of division. Due to this insect-specific feature, it is often difficult to compare early vertebrate and insect embryos in terms of cell-cell interaction and communication. In contrast, spider embryos establish a cell-based organization at the earliest stages of development (Kanayama et al. 2010; Suzuki and Kondo 1995), which facilitates comparisons with vertebrate embryos. Holm conducted bisection-like manipulations of *Agelena* embryos at cumulus-shifting stages, which could result in twinning (Holm 1952). Holm’s manipulations were rather complex; sectioned embryo halves in an egg were rotated opposite to each other and oriented at 180°. This rotation might have prevented the embryo fragments from recombining and then recovering as a single embryo. Using horseshoe crab embryos, Sekiguchi conducted similar field-separation experiments and showed that twinning could occur following field-separation (Sekiguchi et al. 1999). However, to our knowledge, no other investigators have replicated these chelicerate embryological experiments.

**Fig. 4:**
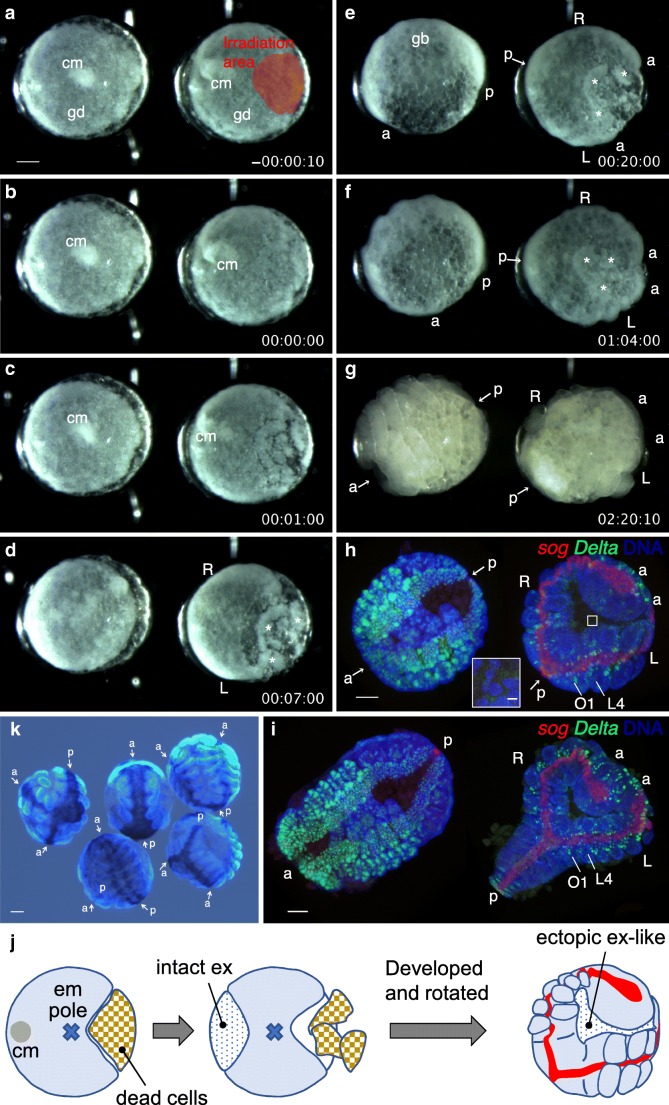
Laser-based ablation of a large region of the embryo in *P. tepidariorum* (A–I) An embryo at the cumulus-shifting stage is irradiated with a laser. (A) The embryo several minutes before laser irradiation (right), together with an untreated sibling embryo (left); the irradiation area is indicated in red. (B–G) Time-lapse recording (days: hours: minutes) after irradiation (B, time 0). Asterisks indicate cell debris that resulted from the laser irradiation. The left (L) and right (R) fields are seen to separately elongate the AP axes. The anterior (a) and posterior (p) ends of the fields or the directions toward them (arrows) are indicated. (H, I) The recorded embryos were fixed within 20 min after image G was taken and were stained for *sog* (red) and *Delta* (green) transcripts, as well as for DNA (blue). Whole (H) and flat (I) mount preparations of the same stained embryos are displayed. The embryos in H are positioned at a similar angle to the live embryos in G. *sog* expression marks the ventral midline region, and *Delta* expression marks differentiating neural cells. In the laser-irradiated embryo, the body axes that cover the first opisthosomal segment (O1) and more anterior segments are duplicated. Note that each of the doubled germ bands is seen to have some bilateral pairs of limb buds developing and the neuroectoderm differentiating in the intermediate regions. The boxed region in (H) is magnified in the inset to show the presence of sparsely distributed, flat nuclei, which are characteristic of extraembryonic cells. Note that the untreated embryo develops much more rapidly than the treated embryo. (J) Schematic interpretation of the consequences of laser irradiation. cm, cumulus; gd, germ disc; gb, germ band; L4, the segment for the fourth walking legs; O1, the first opisthosomal segment; ex, extraembryonic region; em pole, embryonic pole. See also Movie S3. (K) Examples of duplicated body axes caused by laser ablation of large regions of germ discs at the cumulus-shifting stage in another experiment. In four of the five embryos, which were stained for *sog* and *hh* transcripts, the axes appear partially duplicated. The anterior (a) and posterior (p) ends of the fields or the directions toward them (arrows) are indicated. Bars, 100 μm (except for the inset in H, 10 μm)


Recently, using the model spider *Parasteatoda*, we applied laser irradiation to a large region of an embryo that was opposite the cumulus side at the cumulus-shifting stage (Fig. 4A; Supplementary text). As revealed by time-lapse recording (Movie S3), this treatment killed a large proportion of cells in the presumptive ventral region of the embryo (Fig. 4B–D, J) and led the left and right fields of the embryo to separately elongate the AP axes (Fig. 4D–F), followed by formation of a rather normal, bilaterally symmetric pattern in each separated germ band (Fig. 4G–J). Importantly, the region corresponding to the laser-irradiated part of the embryo was occupied by extraembryonic-like cells and acted in concert with surrounding dorsal features (Fig. 4H–J). When the laser-irradiated area is sufficiently small, the embryo can easily resume normal development, as observed for the left embryo in Fig. 2. Blocking cell-cell interactions and communication for a long period or on a larger spatial scale might instead promote independent development of each field. Alternatively, cells close to the ablated sites might act as sources of signals. Hence, axes-doubling phenomena described in chelicerate embryos by Holm and Sekiguchi are relatively easy to reproduce in an emerging spider model system (Fig. 4K), providing a chance to study the flexible regulation of arthropod body axes formation.

Movie S3Laser-based ablation of a large region of the embryo in *P. tepidariorum*. (Left) An untreated embryo. (Right) A sibling embryo that is irradiated with a laser at the cumulus-shifting stage. The irradiation area is indicated in red. After irradiation, time-lapse recording (days: hours: minutes) starts. The left (L) and right (R) fields are seen to separately elongate the AP axes. The recorded embryos were fixed within 20 min after the last image was taken, and were stained for *sog* (red) and *Delta* (green) transcripts, as well as for DNA (blue or white). *sog* expression marks the ventral midline region, and *Delta* expression marks differentiating neural cells. In the laser-irradiated embryo, the body axes that cover the first opisthosomal segment and more anterior segments are duplicated. Note that each of the doubled germ bands is seen to have some bilateral pairs of limb buds developing and the neuroectoderm differentiating in the intermediate regions. Bars, 100 μm. This movie is related to Fig. 4A–I. (MP4 4738 kb)


## Possible mechanisms of self-regulation in arthropod embryos

Compatibility of experimental body axes doubling with modern molecular techniques is a prominent advantage of the model spider *Parasteatoda*. During normal embryonic development of this spider species, overall AP polarity is established through the network activity of Hedgehog (Hh) signaling (Akiyama-Oda and Oda 2010), whereby the *patched* gene (*ptc*), encoding a Hh receptor, acts as a negative regulator. *hh* transcript expression occurs zygotically around the ab-embryonic pole at stage 3 and is localized at the rim of the germ disc at stage 5 (Akiyama-Oda and Oda 2010). Fate mapping confirmed that the peripheral, central, and intermediate regions of the germ disc corresponded to the head, opisthosomal (abdominal), and thoracic regions, respectively, of the future germ band (Hemmi et al. 2018). Knockdown of *hh* by parental RNA interference (pRNAi) causes a lack of anterior fates and predominance of caudal fates, whereas knockdown of *ptc* causes a lack of caudal fates and ectopic occurrence of anterior fates. Axis development in spiders might bear an analogy to segment polarity determination in *Drosophila*. However, although *Wnt8* is required during early opisthosomal development (McGregor et al. 2008; Schönauer et al. 2016), there has been no evidence for a role of Wnt signaling in the formation of overall AP polarity in the spider embryo.

**Fig. 5:**
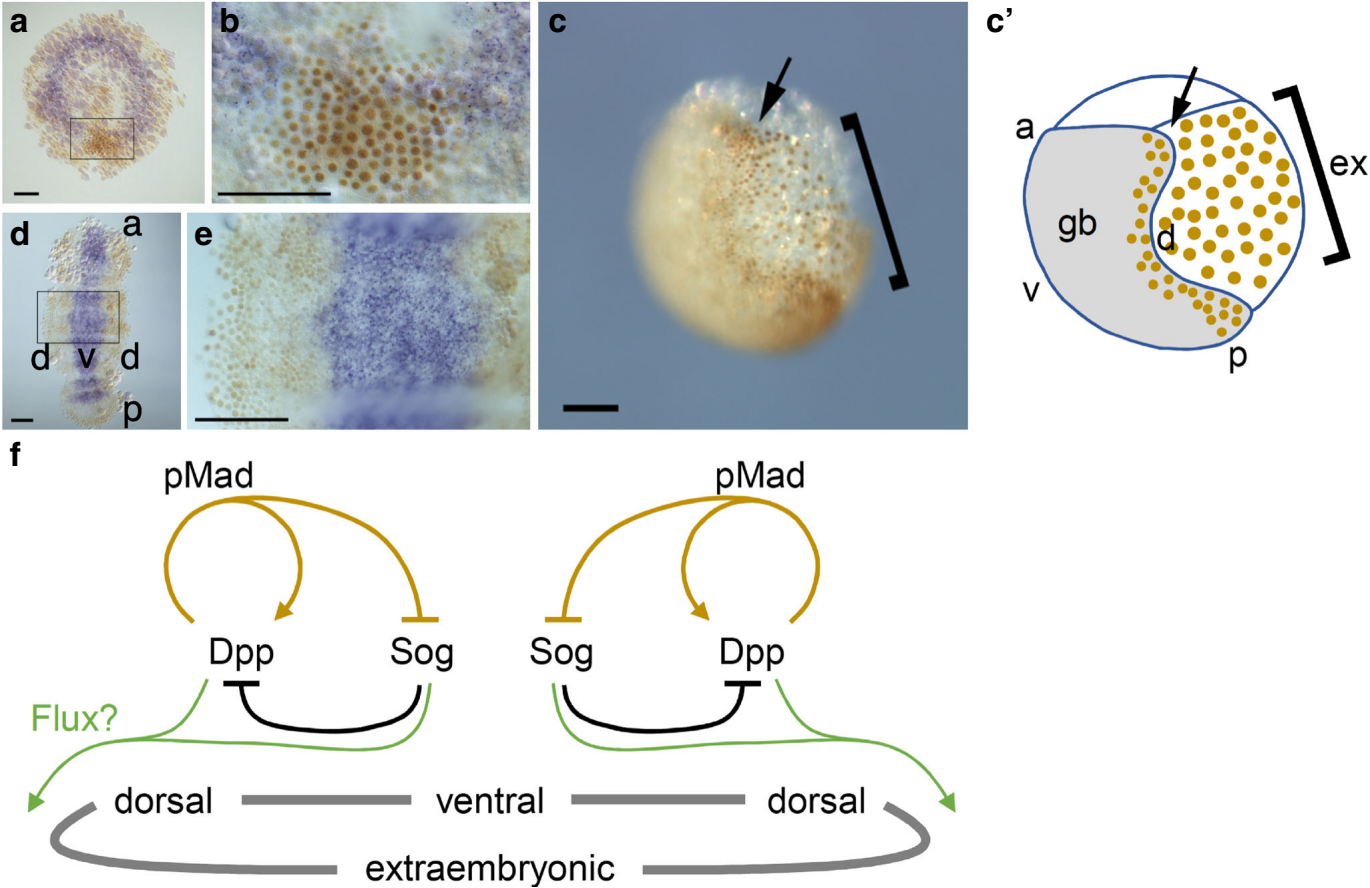
Dynamics of *sog* expression in response to expanding Mad activation in *P. tepidariorum* embryo. Embryos were stained to show the patterns of *sog* transcript expression (purple in A, B, D, E) and pMad protein distribution (brown in A–E). (A, B) A flat-mounted germ disc at the cumulus-shifting stage. The boxed region in (A), which includes the cumulus area, is magnified in (B). In cells receiving Dpp signals, which are associated with the cumulus, Mad is phosphorylated and localized to the nuclei. *sog* expression is repressed in pMad-positive cells. (C, C’) Lateral view of a whole embryo at an early germ band stage (C) and its schematic illustration (C’). A peripheral region of the germ disc that has been specified by the cumulus differentiates into extraembryonic tissue (square bracket), which continues to be pMad-positive. Some embryonic cells (part of the germ band) near the border of the extraembryonic region (arrow) are also pMad-positive. (D, E) A flat-mounted germ band. The boxed region in D is magnified in E. The areas of pMad-positive cells propagate toward the midline of the germ band, restricting the domain of *sog* expression. a, anterior; p, posterior; d, dorsal; v, ventral. All the images are adapted from (Akiyama-Oda and Oda 2006). Bars, 100 μm. (F) Schematic representation of a model depicting regulatory molecular interactions that mediate DV axis specification in *Parasteatoda*. Black and brown lines indicate regulation at the protein and transcription levels, respectively. The two separate network diagrams, mirror image of each other, represent the left and right sides of the embryo, with the DV polarity reflected by Dpp and Sog expression. Green lines indicate the possible flux of the Dpp/Sog complex

The symmetry-breaking movement of the cumulus from the embryonic polar region of the embryo, which corresponds to the center of the germ disc (Fig. 1C), requires the activity of both *ptc* and *Ets4*, the latter of which encodes a transcription factor (Akiyama-Oda and Oda 2010; Pechmann et al. 2017). No molecular signs that help predict the direction of cumulus shifting prior to its onset have been discovered. The cumulus has been characterized as a source of the Decapentaplegic (Dpp) signal (Fig. 5A, B; Akiyama-Oda and Oda 2003, 2006), which mediates induction of extraembryonic fate to initiate the radial-to-bilateral symmetry breaking of the embryo (Figs. 1C and 5C, C’; Akiyama-Oda and Oda 2006). Blocking cumulus shifting by *ptc* pRNAi, but not that driven by *Ets4* pRNAi, coincides with ectopic anterior fates in the embryonic polar region, where ectopic extraembryonic fate is subsequently induced and reveals some DV development along the axis of persistent radial symmetry (Akiyama-Oda and Oda 2010; Pechmann et al. 2017). These observations suggest that cumulus shifting may serve two purposes: on the one hand, it orients the DV axis perpendicular to the AP axis; on the other hand, it brings the signaling center to an area of the field where cells are fully competent to respond to the signal. Although strong signaling activity of the cumulus may ensure robust and efficient progression of DV axis development, no visible cumulus shift is essential for DV axis formation, as suggested by our cumulus extirpation experiment (Fig. 2). The embryonic field appears capable of responding to a faint signaling activity to push forward the programmed DV axis development, but over a longer period of time.

Early response of the embryonic field to the cumulus signal includes phosphorylation and nuclear localization of Mothers against Dpp (pMad), expression of some *GATA* genes, and repression of *short gastrulation* (*sog*) expression (Fig. 5A, B; Akiyama-Oda and Oda 2003, 2006, 2010). *sog* encodes a secreted cysteine-rich protein that is a homolog of vertebrate Chordin, one of the key constituents of Spemann’s organizer. Chordin binds to bone morphogenetic proteins (BMPs, homologs of Dpp), inhibiting their signaling activities and facilitating their diffusion (Reversade and De Robertis 2005; Eldar et al. 2002; Ben-Zvi et al. 2008; Xue et al. 2014). In spiders, during germ band formation and elongation, expansion of the pMad-positive domain occurs concomitantly with restriction of the *sog* transcription domain (Fig. 5D, E) (Akiyama-Oda and Oda 2006). These domains are kept complementary to each other, with the boundary between them presumably progressing from cell to cell. As a result, *sog* transcription becomes confined to the ventral midline, where it mediates specification of the ventral midline and the bilaterally flanking neuroectoderm (Akiyama-Oda and Oda 2006). *sog* expression along the long axis is reminiscent of the axial expression of Chordin in the dorsal tissues of vertebrate embryos. A comparison of the dorsal side of vertebrate embryos with the ventral side of spider embryos reveals the remarkable similarity in expression and function of the homologous *chordin* and *sog* genes (Oda and Akiyama-Oda 2008). This similarity may not be surprising in light of the DV axis inversion hypothesis (Arendt and Nübler-Jung 1994) or the Urbilateria hypothesis (De Robertis and Sasai 1996). However, it is important to note that, except for spiders (Akiyama-Oda and Oda 2006), there has been limited evidence for a functional role of the *sog* gene in specifying the ventral-most tissue, which corresponds to the ventral midline in arthropods. Comparisons between the corresponding sides of vertebrate and insect embryos are complicated by evolutionarily coopted functions of Toll signaling, which contribute to specifying cell fate on the ventral side of syncytial embryos in most insects (Chen et al. 2000; Nunes da Fonseca et al. 2008; Özüak et al. 2014; Sachs et al. 2015). The dependency of ventral fate specification on *sog* seems to have been reduced in some insect sub-lineages.

Mechanisms that regulate the concerted dynamics of Mad activation and *sog* transcription explain, to some extent, the induced doubling of DV pattern elements in two fields that intervene between the intact and grafted cumuli in Holm’s organizer graft experiments. The inhibitory effect of Dpp signaling on *sog* transcription has been evidenced from spider embryos (Fig. 5F; Akiyama-Oda and Oda 2006). This regulation is wide-spread among other bilaterian and even non-bilaterian embryos (Yu et al. 2007; Lowe et al. 2006; Saina et al. 2009; Genikhovich et al. 2015), with the sole exception of some insect embryos (Sachs et al. 2015). Moreover, this regulation might be combined with the positive feedback of Dpp signaling (Fig. 5F), as proposed for BMPs on the ventral side of vertebrate embryos (Reversade and De Robertis 2005). Therefore, it is believed that propagation of Dpp signaling activation from the most distant sites on the spherical surface of the embryo restricts *sog* transcription to two middle areas that will specify ventral midlines.

Carrying out the spider’s organizer experiments requires understanding first how the full range of DV pattern elements can be doubled in a limited space. A similar situation arises also in the field-separation experiments. The problem centers around field self-regulation and scaling of the pattern, which has been rigorously investigated in *Xenopus* embryos. Experimental and theoretical studies have proposed the presence of a directional flux of BMP proteins mediated by Chordin, which allows cell-cell communication over long distances within a continuous field (Ambrosio et al. 2008; Ben-Zvi et al. 2008; Inomata et al. 2013). The BMP/Chordin system possesses positive and negative feedback regulation at protein and transcriptional level, which enables self-regulation and regeneration of morphogen gradients in the field (De Robertis 2009). The BMP/Dpp flux mediated by Chordin/Sog was originally identified in *Drosophila* blastoderm embryos and was suggested to help form a sharp peak of BMP/Dpp activity at the dorsal-most site to induce extraembryonic fate (Eldar et al. 2002; Wang and Ferguson 2005; Mizutani et al. 2005; Shimmi et al. 2005). Notably, the same might be applied to spider embryos (Fig. 5F). Phenotypes of spider embryos knocked down for *sog*, however, have provided no evidence supporting delivery of Dpp to the dorsal side of the embryo to induce extraembryonic fate. There is, nonetheless, an intriguing possibility that in field-separation experiments, Sog might shuttle Dpp to the ab-cumulus (or laser-irradiated) side of the separated fields to promote extraembryonic induction, which is potentially facilitated by self-enhancement of Dpp signaling activity. This possibility should be tested by future studies, together with alternative explanations of ectopic extraembryonic induction. One of them, for example, is that cells at the injured sites might gain organizer-like signaling activity in response to changes in chemical and physical conditions.

A recent experimental and theoretical study using eggs of the milkweed bug *Oncopeltus fasciatus*, a hemipteran insect, suggested that simulations of a minimal Sog/BMP network could reproduce opposing Sog and BMP gradients in a field (Sachs et al. 2015). Intriguingly, this study also showed that the same network enabled separated ventral and dorsal half fields to self-regulate, a phenomenon mimicking bisection-induced twinning reported by Klaus Sander for the eggs of the hemipteran leaf hopper *Euscelis plebejus* (Sander 1971). A key regulation assumed in the network is inhibition of *sog* transcription by BMP activity, a feature shared by the spider embryo but not by some other insect embryos as mentioned above. The spider and hemipteran embryos differ in terms of what serves as a DV axis polarity cue in the field: displacement of a Dpp signaling center in the cellular field of spiders, versus a nuclear gradient of maternal Toll signaling activity in the syncytial field of insects. In the latter, the spatially biased input from Toll signaling is believed to affect BMP signaling activity. Although there is an apparent difference in the polarization process, it remains to be determined whether and/or how the mechanisms underlying the self-regulatory capabilities of the hemipteran and spider embryonic fields are related to each other from a molecular and theoretical viewpoint. In addition, it should be noted that the hemipteran simulation study only considered ventral-dorsal bisection, even though the twinning in Sander’s experiments was achieved irrespectively of the plane of bisection (Sander 1971).

Presence of an axial organizer and self-regulation of embryo fragments and embryonic cell aggregates have been documented in the non-bilaterian cnidarian *Nematostella vectensis* (Fritzenwanker et al. 2007; Kraus et al. 2007; Saina et al. 2009; Genikhovich et al. 2015; Kraus et al. 2016; Kirillova et al. 2018)*.* These studies, based on developmental molecular data, have provided models for how bilaterian and non-bilaterian embryos can be compared. Integrating with the non-bilaterian data, the self-regulatory properties of embryonic fields in some arthropods might reflect the ancestral state of the mechanisms of bilaterian body axes formation. In arthropod research, access to self-regulation phenomena has been limited for various reasons. There are technical obstacles to embryological manipulations in *O. fasciatus* (Sachs et al. 2015), and little molecular work has been attempted in *E. plebejus* or its kin. Therefore, development of appropriate model systems is a critical step in unraveling the mechanisms of embryonic twinning and self-regulation.

## Benefits of the spider model system

Spiders are currently the only arthropods whose use allows us both to induce duplication of bilaterian body axes via embryological manipulations and adopt molecular approaches to investigate mechanisms underlying embryonic events. Among many spider species, *P. tepidariorum* stands out for its suitability in experimental embryology, as a result of its physiology as well as researchers’ efforts.

A mated *Parasteatoda* female adult lays approximately 200 eggs in an egg sac (they develop simultaneously) and has regular cycles of egg production (each cycle takes 4 to 6 days). Morphological development of embryos is easily observed through the chorion, after placing eggs in oil. These natural features of the spider have been effectively combined with a gene knockdown technique that uses pRNAi (Akiyama-Oda and Oda 2006) to facilitate functional screening for genes involved in regulation of embryonic phenomena of interest. Indeed, this technique allows even a small laboratory to identify genes responsible for cell-cell interactions and communication that result in body axes specification (Akiyama-Oda and Oda 2010; Kanayama et al. 2011; Pechmann et al. 2017). Such approach allowed for the unexpected discovery of early *hh* and *ptc* functions. Availability of a pre-screen step using microarrays or other gene expression profiling techniques would further reduce reliance on candidate-gene strategies (Kanayama et al. 2011; Pechmann et al. 2017). A microinjection-based gene knockdown technique, embryonic RNAi, is also applicable to *Parasteatoda* embryos, thus enabling gene function analysis in cell clones (Kanayama et al. 2011). As with *Drosophila* genetic mosaics, this technique may help us obtain molecular clues about the interactions occurring among cells in a field.

The availability of genome and transcriptome sequences of *P. tepidariorum* (Oda et al. 2007; Posnien et al. 2014; Schwager et al. 2017; Sasaki et al. 2017; Iwasaki-Yokozawa et al. 2018) facilitates genome-scale studies to identify and characterize developmental gene regulatory interactions by combining sequencing-based gene expression profiling and gene knockdowns (unpublished, Y.A. and H.O). There is also an increasing variety of high-throughput sequencing methods (e.g., single-cell RNA-seq, ChIP-seq, and ATAC-seq), which have revolutionized developmental biology research (Treutlein et al. 2014; Jaitin et al. 2014; Rotem et al. 2015; Cusanovich et al. 2015, 2018; Sebé-Pedrós et al. 2018). These methods could be applied in spiders on pools of siblings from egg sacs.

A further benefit comes from the simple geometry of the cell-based embryo, which is convenient for gene expression data presentations and mathematical descriptions of pattern formation. The radial symmetry is set up in a disc of mono-layered epithelial cells, the germ disc, which covers the upper spherical surface of the embryo. The germ disc and subsequent forms of embryonic cells allow semi-flat specimen mounting for microscopic observation. Gene expression dynamics associated with AP and DV pattern development can be displayed in a two-dimensional framework (Akiyama-Oda and Oda 2006; Hemmi et al. 2018). Multi-color fluorescence in situ hybridization can be applied for gene expression analysis, facilitating quantitative studies (Akiyama-Oda and Oda 2016; Hemmi et al. 2018). Cell labeling and tracking can be combined with gene expression analysis to investigate the relationship between cell behaviors and gene expression dynamics (Kanayama et al. 2011; Hemmi et al. 2018). Thus, the *Parasteatoda* model system may have suitable and compatible features for experimental and theoretical studies of pattern formation involving cell proliferation, cell movement, and cell-cell interaction.

Despite the strong potential of the *Parasteatoda* model system, neither transgenesis nor gene editing have been successful in this organism, contrasting the case in other emerging model arthropods (Pavlopoulos et al. 2004; Pavlopoulos and Averof 2005; Nakamura et al. 2010; Kontarakis et al. 2011; Watanabe et al. 2012; Kato et al. 2012; Gilles et al. 2015; Kao et al. 2016; Kumagai et al. 2017). Another disadvantage is posed by *Parasteatoda*’s largely duplicated genome with potentially redundant functions (Schwager et al. 2017; Leite et al. 2018). Moreover, the number of available antibodies for specific proteins is limited, preventing deeper analyses of molecular interactions and mechanisms. Although, in general, cell culture systems facilitate cell biological and biochemical studies, there are no spider-derived cell lines available. Thus, continuing efforts are required to overcome these technical limitations.

## Conclusions

The significance of the pioneering work by Holm, which demonstrated experimental duplication of the spider body axes, commands wider appreciation. The two concepts of developmental biology, organizer and self-regulation, which were described in vertebrate embryology, are similarly applied in the development of spiders. They explain the induced doubling of the bilaterian body axes in experimental perturbations. Although the organizers in both systems are evidently not homologous to each other, the general mechanisms underlying inducible axes duplication and self-regulation might share a common origin. To pursue this working hypothesis, an in-depth comparative analysis of the mechanisms using appropriate model systems in vertebrates and arthropods should be performed. Among an increasing number of model arthropod species, the common house spider *P. tepidariorum* allows replication of embryological twinning experiments, as well as cellular, molecular, and theoretical work on the mechanisms of body axes formation. The merits and nature of the *Parasteatoda* model system could contribute to a better understanding of the basic principles of bilaterian body axes development and evolution.

## Electronic supplementary material

ESM 1 (PDF 100 kb)
