# Supplementary material for: Experimental duplication of bilaterian body axes in spider embryos: Holm’s organizer and self-regulation of embryonic fields
Source: Dev Genes Evol. 2019 Apr 10;230(2):49–63. doi: 10.1007/s00427-019-00631-x (PMC7128006; doi:10.1007/s00427-019-00631-x)
Supplement: Supplementary file 4 — (PDF 100 kb) [file 427_2019_631_MOESM1_ESM.pdf]

Supplementary text for:

Experimental Duplication of Bilaterian Body Axes in Spider Embryos: Holm's Organizer and Self-regulation of Embryonic Fields

Development Genes and Evolution

Hiroki Oda[1, 2, *], Sawa Iwasaki-Yokozawa[1], Toshiya Usui[3], Yasuko Akiyama-Oda[1, 4]

[1]Laboratory of Evolutionary Cell and Developmental Biology, JT Biohistory Research Hall, 1-1 Murasaki-cho, Takatsuki, Osaka, 569-1125, Japan

[2]Department of Biological Sciences, Graduate School of Science, Osaka University, Osaka, Japan

[3]Nagoya Minami High School, Aichi, Japan

[4]Microbiology and Infection Control, Osaka Medical College, Osaka, Japan

*Corresponding author: Hiroki Oda (hoda@brh.co.jp)

Materials and methods

Cumulus transplantation

Hasarius adansoni eggs from an egg sac were dechorionated with 100% commercial bleach for about 3-4 min and rinsed several times in distilled water. After removing the water, the embryos were immediately transferred one by one onto double-sticky tape on the bottom of a dish and covered with halocarbon oil 700 (Sigma-Aldrich). To make tissue transfer needles, glass capillaries (Cat. No. 2-000-075, Drummond Scientific Company) were pulled using a puller (PN-3, Narishige). The tip of each transfer needle was broken using forceps so that the tip inner diameter was approximately 10–20 μm. Using the transfer needle connected to a suction tube, cumuli were transferred between sibling embryos. The dechorionation step was not essential for successful transplantation of cumuli but was required for clear visualization of embryonic development.

Laser ablation

Parasteatoda tepidariorum eggs from an egg sac were dechorionated with 100% commercial bleach for about 3–4 min and rinsed several times in distilled water. After removing the water, the embryos were immediately transferred one by one onto

double-sticky tape on the indented region of a specially designed glass slide and covered with halocarbon oil 700 (Sigma-Arldrich), in a way similar to the microinjection procedure previously described (Kanayama et al. 2010). For laser ablation, an upright microscope (BX50, Olympus) equipped with the XYClone 20× laser-objective (Hamilton Thorne) was used. In the experiment shown in Fig. 2 and Movie S1, 12 infrared laser pulses (1460 nm, 300 mW × 3 ms) were applied to various sites in the target area, whereas in the experiment shown in Fig. 4 and Movie S3, 23 laser pulses were applied. In each experiment, a pair of untreated and laser-treated sibling embryos was time-lapse recorded using a stereomicroscope (M165C, Leica) equipped with a color CMOS camera (WRAYCAM-G200, WRAYMER).

Embryo staining

Immunostaining for β-catenin was performed as described previously (Oda et al. 2007). Briefly, embryos were fixed in a two-phase solution of heptane and 5.5% formaldehyde in PEM (100 mM PIPES, 1 mM EDTA, 2 mM $MgSO_4$, pH 6.9), followed by manual removal of the vitelline membrane using forceps in phosphate-buffered saline with 0.1% Tween-20 (PBS-T). The embryos were washed with PBS-T, blocked with 5% skim milk in PBS-T and then incubated with a commercially available rabbit anti-β-catenin antiserum (C2206, Sigma-Aldrich) at a 1:1000 dilution overnight at 4°C. Donkey anti-rabbit IgG labeled with Cy5 (Chemicon) was used as secondary antibody at a 1:200 dilution. Samples were counterstained with 1 U/mL phalloidin-fluorescein (Molecular Probes) and 0.5 mg/mL DAPI (Sigma-Aldrich). Stained samples were examined with an Olympus IX71 microscope equipped with a cooled CCD camera (CoolSNAP HQ, Roper Scientific) controlled by the software Metamorph version 6.1 software (Universal Imaging).

Multi-color Fluorescence *in situ* hybridization for *P. tepidariorum sog* and *Delta* transcripts was performed as described previously (Akiyama-Oda and Oda 2016).

References

Akiyama-Oda Y, Oda H (2016) Multi-color FISH facilitates analysis of cell-type diversification and developmental gene regulation in the *Parasteatoda* spider embryo. Dev Growth Differ 58:215-224

Kanayama M, Akiyama-Oda Y, Oda H (2010) Early embryonic development in the spider *Achaearanea tepidariorum*: microinjection verifies that cellularization is complete before the blastoderm stage. Arthropod Struct Dev 39:436-445

Oda H, Nishimura O, Hirao Y, Tarui H, Agata K, Akiyama-Oda Y (2007) Progressive activation of Delta-Notch signaling from around the blastopore is required to set up a functional caudal lobe in the spider *Achaearanea tepidariorum*. Development 134:2195-2205
